# A systematic scoping review of the evidence for consumer involvement in organisations undertaking systematic reviews: focus on Cochrane

**DOI:** 10.1186/s40900-016-0049-4

**Published:** 2016-12-21

**Authors:** Richard F Morley, Gill Norman, Su Golder, Polly Griffith

**Affiliations:** 1 Cochrane, St Albans House, 57-59 Haymarket, London, SW1Y 4QX UK; 2 Division of Nursing, Midwifery and Social Work, School of Health Sciences, Faculty of Biology, Medicine and Health, University of Manchester, Jean McFarlane Building, Oxford Road, Manchester, M13 9PL UK; 3 Department of Health Sciences, University of York, Heslington, YO10 5DD UK; 4 Consumer representative, Cochrane Pregnancy and Childbirth, York, UK

**Keywords:** Consumer, Involvement, Systematic Reviews, Organisations, Impact

## Abstract

**Plain English summary:**

Cochrane is the largest international producer of systematic reviews of clinical trial evidence. We looked for published evidence that reports where consumers (patients and the public) have been involved in Cochrane systematic reviews, and also in reviews published by other organisations.

We found 36 studies that reported about consumer involvement either in individual systematic reviews, or in other organisations. The studies showed that consumers were involved in reviews in a range of different ways: coordinating and producing reviews, making reviews more accessible, and spreading the results of reviews (“knowledge transfer”). The most common role was commenting on reviews (“peer reviewing”). Consumers also had other general roles, for example in educating people about evidence or helping other consumers. There were some interesting examples of new ways of involving consumers. The studies showed that most consumers came from rich and English speaking countries. There was little evidence about how consumer involvement had changed the reviews (“impact”). The studies found that consumer involvement needed to be properly supported.

In future we believe that more research should be done to understand what kind of consumer involvement has the best impact; that more review authors should report how consumers have been involved; and that consumers who help with reviews should come from more varied backgrounds.

**Abstract:**

**Background**

Cochrane is the largest international producer of systematic reviews, and is committed to consumer involvement in the production and dissemination of its reviews. The review aims to systematically scope the evidence base for consumer involvement in organisations which commission, undertake or support systematic reviews; with an emphasis on Cochrane.

**Methods**

In June 2015 we searched six databases and other sources for studies of consumer involvement in organisations which commission, undertake or support systematic reviews, or in individual systematic review processes. All types of reports and evaluations were eligible. Included studies were combined in a narrative synthesis structured by the level of evaluation and the type of involvement.

**Results**

We identified 36 relevant studies. Eleven of these were evaluations at the level of a whole organisation; seven of these studied consumer involvement in Cochrane. Ten studies examined individual Cochrane review groups. Twelve studies reported on individual reviews; only two of these were Cochrane reviews. Finally, three studies were themselves syntheses of other studies. The included studies reported varying levels of consumer involvement across a wide range of activities related to review design and conduct. These included activities such as priority setting and outcome definition as well as review-specific roles such as acting as peer referees and producing plain language summaries. The level of satisfaction and awareness of impact was generally higher in studies focused on individual Cochrane review groups than in the organisation-wide studies.

**Conclusions**

There was evidence of highly variable levels and types of consumer involvement within and beyond Cochrane, but limited evidence for what makes the most effective methods and levels of involving consumers in review production. Where evidence of impact was found at the level of individual reviews and review groups it underlined the need for properly resourced and supported processes in order to derive the greatest benefit from the lived experiences of consumers who are willing to be involved. Where reviews do involve consumers, their contribution to the final result could be more clearly identified. More rigorous evaluations are needed to determine the best approach to achieving this.

**Trial registration**

Not applicable.

**Electronic supplementary material:**

The online version of this article (doi:10.1186/s40900-016-0049-4) contains supplementary material, which is available to authorized users.

## Background

There is increasing acceptance of the need to involve patients and members of the public (consumers) in all types of health research. This includes systematic reviews, which help shape health policy and practice. There is existing research on the impact of involving consumers in systematic reviews both at the level of the individual review [1] and at an organisational level [2].

Cochrane is a global independent network of researchers, professionals, patients, carers, and people interested in health and is the largest international producer of systematic reviews involving 37,000 contributors from more than 130 countries. Cochrane reviews are overseen by Cochrane Review Groups (CRGs) of which there are presently 53, organised by health condition or setting. Cochrane is committed to involving consumers in the production and dissemination of systematic reviews. Healthcare consumers who use Cochrane evidence are generally patients, carers and family members, or people interested in remaining healthy who are seeking information about a health condition or treatment for personal use. Cochrane’s Consumer Network (CCNet) comprises over 1500 healthcare consumers and others, including members of patient advocacy groups and people with an interest in the organization of healthcare, such as practitioners who support patient participation in health research.

This systematic scoping review aims to document and analyse the most recent evidence on involvement in systematic reviews at multiple levels of Cochrane and to identify models used in other organisations which commission or produce systematic reviews. This forms part of a broader review of the structures and functions of Cochrane that includes consumer involvement. Where possible we sought to identify the impacts of particular types of consumer involvement on outputs and processes. Research on consumer involvement in individual systematic reviews was also gathered and used to allow comparisons between involvement in individual and organisational level reviews.

## Methods

### Objective

To conduct a systematic scoping exercise to evaluate the evidence base on consumer involvement in organisations which commission, undertake or support systematic reviews; with an emphasis on Cochrane.

### Inclusion criteria

Any study which evaluated or reported on consumer involvement in an organisation which commissions, undertakes or otherwise supports systematic reviews, or which reported on consumer involvement in an individual systematic review process (or processes) was eligible for inclusion.

### Exclusion criteria

We excluded studies which reported on consumer involvement in research which did not explicitly include systematic reviews. This included studies of priority setting exercises where this was not related to the undertaking of systematic reviews.

### Search methods

We searched the following databases and other electronic sources in June 2015:

- CINAHL Plus
- MEDLINE In-Process & Other Non-Indexed Citations and MEDLINE
- Embase
- Cochrane Methodology Register (CMR)
- HMIC Health Management Information Consortium
- ProQuest Dissertations & Theses: UK & Ireland

The searches were date-limited to records from 1990 onwards and were designed by an information specialist. Full search strategies are provided in the Additional file 1: Table S1, S2, S3. They included search terms related to Cochrane.

Records retrieved were independently assessed by two researchers. Potentially relevant records were obtained as full texts. Full text assessment was also carried out by two independent researchers. One researcher also searched the abstracts of Cochrane Colloquia. We contacted authors/experts in the field in order to identify full texts of potentially relevant studies identified in this way but did not explicitly contact the consumer network. References from all identified studies and other relevant summaries and bibliographies were also checked to identify additional studies.

### Data extraction and Synthesis

One researcher extracted data into structured tables. Data were extracted on the following:

- Bibliographic information
- Country where data were collected
- Organisation, group or topic evaluated
- Methodological or geographical focus
- Study methodology
- Individuals and activities surveyed or evaluated
- Consumer characteristics
- Results of consumer involvement
- Results of evaluation
- Impact of consumer involvement

Studies were grouped by the level at which the evaluation was performed (organisational, individual Cochrane Review Group (CRG), or individual systematic review(s)). Organisational studies were further grouped by whether the evaluation was focused on Cochrane, another organisation or on multiple organisations. Studies of individual CRGs were grouped by the CRG evaluated. Syntheses of studies were not extracted as individual studies but were used to ensure that all individual studies were identified and included.

A narrative synthesis was produced, structured by the study groupings identified. Themes of types of involvement and impacts were identified, as were perceived barriers to involvement. Emphasis was given to those studies which evaluated the impacts of consumer involvement. Consumers’ perceptions of their involvement were also highlighted.

## Results

**Fig. 1:**
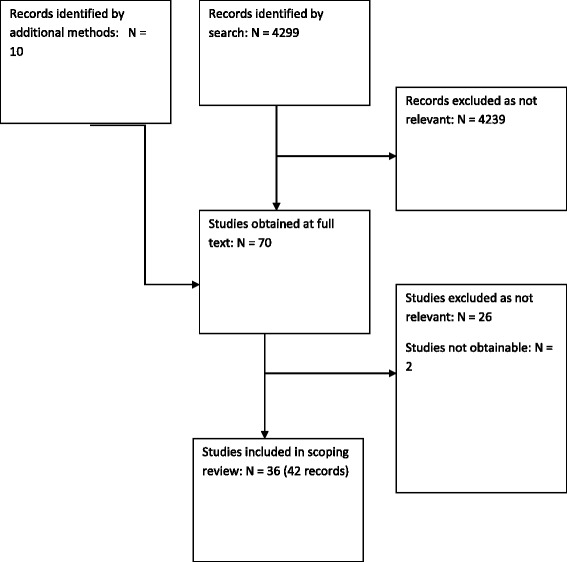
Flow diagram of identified records and included studies

We identified a total of 4299 records from the database searches. Sixty of these were identified as potentially relevant and ordered as full texts. Searching Cochrane abstracts, contacting authors and experts in the field and reference checking identified an additional 10 records. 42 records referred to 36 relevant studies and were included, 26 were excluded and 2 records have proved unobtainable (see Additional file 2: Appendix 1). A total of 36 studies reported in 42 publications were included (Fig. 1).

### Types of included studies

We identified four types of reports (Table 1):

**Table 1:** summary of identified studies

Number of studies	Organisational level evaluations	CRG case reports	Individual reviews	Syntheses/summaries
Total	11	10	12	3
Cochrane	7	NA	2	NA
Non-Cochrane	4	NA	10	NA

1. Surveys and other evaluations of consumer involvement at an organisational level in Cochrane or another organisation
2. Case reports, surveys or other evaluations of consumer involvement in a single CRG
3. Case examples of consumer involvement in individual systematic reviews (Cochrane and non-Cochrane)
4. Syntheses or summaries of consumer involvement in individual systematic reviews

### Organisational level evaluations (Additional file 1: Table S1)

Seven organisational level evaluations focused on the Cochrane collaboration [3–9]; three focused on other organisations [10–12] while one evaluated multiple organisations including Cochrane and some individual CRGs [13]. A final study looked at the views of consumers from external organisations on Cochrane reviews from the perspective of the German HTA (IQWiG) [14].

### Reports from individual CRGs (Additional file 1: Table S2)

The 10 case studies identified related to only four CRGs. These were the Pregnancy and Childbirth Group (4 studies), [15–17] the Skin Group (2 studies), [18, 19] the Musculoskeletal Group (3 studies), [20–22] and the Haematological Malignancies Group (1 study) [23]. Study dates ranged from 1998 to 2013. Groups were based in the UK, USA, Canada and Germany. One of these studies represented the only comparative design identified – this was a historical comparison of two methods of involvement [15].

### Individual review case studies (Additional file 1: Table S3)

We identified a total of 12 case studies of consumer involvement in 13 individual systematic reviews [24–36]. Only two of the case examples were Cochrane reviews [27, 29]. We also identified three syntheses or summaries of case examples [1, 2, 37]. The great majority of the case studies included in these research syntheses were independently identified by our search strategy. We therefore took the decision not to extract or further analyse the syntheses but to include any remaining studies as case examples. This represented a protocol deviation but we believe that in the context of a scoping review, it maximised the available information.

### Differences between the study types identified

Studies of involvement at different levels took different approaches to the types of data sought and the ways in which both types of involvement and impacts were defined and characterised. This was partly a consequence of the breadth of possible involvement surveyed but also reflected the particular focus of study authors.

Organisational level studies were more likely to report hard data on numbers of consumers involved at specific stages of the review production process, whilst studies of individual groups or reviews, perhaps unsurprisingly, were more likely to report qualitative data on the views of consumers, reviewers and other stakeholders about their level of involvement and its perceived impacts. Most likely to report a concrete change in the finished research were studies of individual reviews, perhaps because the process was easier to trace for a single piece of research.

Reports from individual review groups or of involvement in individual reviews were also extremely likely to be positive about involvement and impact from both the consumer and the researcher/organisational perspective. This is perhaps also unsurprising, and is probably indicative of the fact that these publications are likely to derive from groups of researchers and consumers which have a high level of commitment to involvement. The suggestion that this published/reported literature may be self-selecting is supported by the more ambivalent levels of confidence in impact identified by Cochrane-wide surveys which included almost all the review groups.

### Types of involvement

Most of the studies had a general focus. Earlier studies tended to address whether consumers were involved in any capacity, and often to report this as a perceived impact in itself. However, later studies reported in more detail on the types and depth of involvement. The types of involvement which were documented could be broken down into four main categories: formal roles in review production and co-ordination, roles in review production, roles in increasing the accessibility of review, and roles in knowledge transfer. Individual contribution types are shown in Table 2. This also lists the perceived impacts of consumer involvement in terms of the final reviews produced. Broader impacts are also discussed later. A minority of studies focused on a specific activity such as outcome identification, priority setting or peer reviewing, either across organisations or within a Review Group. All contributions and roles contained in the table represent reported involvement; roles which are desirable but not reported as undertaken are not included.

**Table 2:** Consumer roles and identified impacts on outputs

Consumer roles (defined by role in review or organisation)	Consumer activities in review process	Consumer roles in increasing accessibility of reviews	Consumer roles in knowledge transfer	Impacts identified
Membership of editorial teams	Hand searching	Plain language summary preparation	Coordination of workshops, seminars and presentations on CRG activities during consumer organisation conferences	Use of consumer-oriented/more relevant outcomes
Consumer coordination	Obtaining information	Removal of unfriendly jargon from synopses & other documentation	Participation in working groups	Changes in language – greater accessibility
Review authorship	Peer review of protocols and reviews	Collaboration with external consumer organisations to publish synopses	Dissemination	Informing of/altering methodology
Peer reviewer	Priority setting	Translation	Raising awareness of evidence-based healthcare	Adding “depth” to the review
Advisory group member	Incorporation of patient-derived outcomes	Publication of synopses/other CRG documentation in languages other than English	Identifying the needs for non-professional consumer involvement	Better prioritisation of review topics
Stakeholder (various stages)	Input into relevance of review question		Recruiting others	Increased relevance of review
Member of the public			Peer mentoring	Increased timeliness
			Training other consumers	Reduction in bias
			Input into funding bids	Broader and more inclusive literature searches
			Developing partnerships with other relevant organisations	More clearly defined research questions
			Contributions to newsletter	Identification of additional included studies
			Preparing decision aids for patients	Increased depth, relevance and usefulness of review
				Indirect benefits to all involved

Both of the individual Cochrane reviews reported high levels of consumer involvement throughout the review processes [27, 29]; levels and types of involvement in the non-Cochrane reviews were more variable. Many were conducted by or in partnership with the UK EPPi Centre or the Social Care Institute for Excellence (SCIE) and covered public health topics. The level of reported involvement varies greatly: from a consumer acting as a principal investigator; through co-authorship; heavily involved advisory groups; to a single conference with users supported by some additional contact. These case examples have almost all been included in at least one published synthesis (Boote 2011, Boote 2012, Carr 2007) and are not the primary focus of this work [1, 2, 37]. They are not, therefore analysed in detail but are detailed for the interested reader in Additional file 1: Table S3.

### Levels of involvement

#### Organisational and review process activities

Organisational roles undertaken by consumers in Cochrane included as member of editorial teams, consumer co-ordinator, review author and peer reviewer. By far the most common of these was peer reviewer of protocols and reviews [6]. Reflecting this, peer review was one of the key activities in the review process, although it occurs late on in the review production. Activities which occur before a review begins, such as priority setting, question setting, or outcome determination were much less frequently reported, although increasingly regarded as important. Other activities occurring earlier in the review process included hand searching and “obtaining information”. It was notable that the Skin Group (one of the four CRGs with published case studies) reported that there were high levels of co-authorship among consumers, which may be reflective of their generally high levels of consumer activity and satisfaction [19].

In non-Cochrane organisations consumers were also members of advisory boards [13] or were identified as stakeholders with roles throughout and across the review process [10].

#### Roles in increasing accessibility

A larger number of roles for consumers which we identified could be classed as increasing accessibility of reviews or knowledge transfer, although these inevitably overlap to some degree with review processes and with each other. Activities included reviewing and preparation of plain language summaries, and editing of language in both these and other synopses. They also included translation activities – both translation of full reviews and preparation of synopses in languages other than English. Collaboration with external consumer organisations to publish synopses can also be included with this type of activity.

#### Review promotion activities and knowledge transfer

Knowledge transfer was identified as the largest class of consumer-related activity. Consumers were documented as being involved across a very wide range of activities which promoted the role of reviews and research more generally, rather than involvement with a single review or set of reviews (Table 2). This included participation in working groups and partnership development work, conference-based activities, educational, outreach or awareness raising for reviews and evidence-based healthcare more generally. In some cases this also included preparing decision aids for patients. Consumers were also documented as writing for other media and, crucially, providing different types of support and training for fellow consumers and facilitating their recruitment. In some cases contributions to funding applications were documented. These activities were particularly documented in the very active consumer involvement of the Canadian Health Technology Assessment (HTA) and the Cochrane Musculoskeletal Group [10, 20–22].

#### Levels of consumer involvement: Cochrane

Some of the Cochrane-wide evaluations used surveys of CRGs or audits of data returned by CRGs for other purposes, [7, 8] while others directly surveyed or interviewed consumers and people in other key roles in CRGs [3–6]. Earlier reports focused on whether consumers were involved in any capacity, finding that the majority of CRGs (25/33; 79% response rate) had consumer involvement and that many of the others were planning to introduce involvement [4]. Only 6 CRGs described themselves as unconvinced about the merits of consumer involvement. These early studies also reported a range of roles for consumers within CRGs [3]. However, as late as 2009 a substantial minority of CRGs stated that they did not involve consumers (8/47 responses; 90% response rate) or did not respond to the question (4/52) [6].

Unsurprisingly all the case studies of CRGs we identified showed high or increasing levels of involvement because they were produced by self-selecting review groups. An interesting case study of methods to increase involvement was conducted by Skoetz (2005) who reported on a pilot project to establish a consumer network for the Haematological Malignancies CRG based in Germany [23]. This was the only case study conducted in a non-English speaking country. This project involved the provision of workshops covering topics such as research methods followed by ongoing engagement. This increased participation in both this CRG and in two other oncology CRGs; positive consumer reviews also increased with responses indicating that people felt patient concerns were very influential in the review process. The CRG notes that running a focus group on a review produced better and more comprehensive feedback than “just sending out forms”.

#### Level of consumer involvement: Beyond Cochrane

We identified one relatively recent multi-organisation study. Kreis (2013) conducted in-depth interviews with key informants, combined with reviews of organisational websites across 17 US-based and international organisations involved in the production of systematic reviews; Cochrane and the Musculoskeletal and Pregnancy and Childbirth Groups were included [13]. Seven organisations reported that they routinely involved consumers in reviews. These were the Campbell and Cochrane collaborations, the two CRGs, the Agency for Healthcare Research and Quality (AHRQ) and the Oregon and Johns Hopkins Evidence-based Practice Centres (EPC). Other organisations reported occasional involvement in reviews or regular involvement in other aspects of their operations.

Types of involvement included priority setting (extensive involvement being reported by AHRQ and its EPCs). Other involvement included suggestion of topics and advice on key review questions. Consumers sat on advisory groups, commented on review and protocol drafts and in some cases acted as review co-authors.

Evaluations from individual organisations other than Cochrane were conducted by a Canadian HTA-style organisation (Keown 2008), the US Drug Effectiveness Review Project (McDonagh 2006) and the UK MRC Clinical Trials Unit (Vale 2012a) (which included all studies of which a minority were systematic reviews) [10–12]. Both Keown and McDonagh, using very different models of engagement, identified significant and substantive changes to the final reviews as a consequence of public involvement.

An audit of involvement by 80 stakeholders over four years and 22 reviews by the Canadian HTA organisation documented five stages of possible involvement from topic consultation to determine the research question through to drafting, production and dissemination of the final report [10].

A very different model of consumer involvement was evaluated by McDonagh (2006) [11]. All 26 drug class reviews produced by the US Drug Effectiveness Review Project (DERP) were made publicly available for comment through the organisational website.

An organisation-wide survey of consumer involvement in all types of research was carried out by the UK Medical Research Council (MRC) clinical trials unit [12]. Systematic reviews made up only a minority (*n* = 23, 17%) of studies. The number with consumer involvement was not reported but two examples of useful consumer involvement were highlighted. These are included in the individual systematic review case examples.

### Evaluation of specific consumer roles

Cochrane organisational evaluations which focused on particular aspects of involvement looked at involvement of consumers in incorporating patient-derived outcomes into the review processes [4], the use of consumer refereeing for all protocols and reviews [8]; and levels and types of involvement in priority setting [5]. The role of the CCNet was explored by one study as part of a more general evaluation [7]. One study looked specifically at consumer involvement in a non-Western country, examining the establishment of a multi-disciplinary group of consumers in China [3, 8].

#### Specific roles: patient-defined outcomes

Identification of relevant outcomes was one of the key activities identified by both Cochrane-wide and CRG-specific case studies. An early study by Kelson (1999) identified CRGs’ interest in patient defined outcomes [4]. Thirty-three of 42 CRGs returned completed questionnaires. Of these just over half (58%) had discussed patient-defined outcomes while around a third (36%) had liaised with consumers or consumer organisations about them and a further two had identified organisations to contact.

Identification of relevant outcomes was identified by Ghersi (2002) as an important impact of consumer involvement in the Breast Cancer Group and by Kelson (1999) across CRGs [3, 4]. It was not specifically identified by other general surveys as being a key contribution, but may still have been included in the high levels of comments and contributions to protocols and reviews. Case studies from the Pregnancy and Childbirth and Musculoskeletal CRGs, while not specifically focused on this issue, identified suggestions of relevant outcomes as one of the main contributions of consumers to reviews [15–17, 20, 38].

#### Specific roles: priority setting

Nasser (2013) identified limited consumer involvement in formal priority setting across the CRGs and other Cochrane groupings. 29/52 groups (78% response rate) had a process to inform prioritisation. Stakeholder involvement was reported in all except two of those reporting a process; not all of these involved consumers. Fourteen groups appeared to involve consumers in some way in the process; only one of these involved the public and the press. Only one formal appeal process for the challenging of prioritisation decisions was identified [5].

Priority setting was identified (by other papers which did not specifically focus on this as a discrete topic) as something to which consumers contributed. Five CRGs identified topic prioritisation as an activity undertaken by consumers in Wale (2010) and it was identified as a positive impact of involvement by Kelson (1999) [4, 6]. Two of the four CRGs for which case studies were identified reported priority setting as one of the activities undertaken by consumers (Pregnancy and Childbirth Group and Musculoskeletal Group) [16, 17, 20–22].

Bastian (2011) took a novel approach to involvement in priority setting. She used a web-based survey of employees of the German HTA and members of CCNet and another international email lists [14]. Respondents were asked to evaluate the level of interest of Cochrane review summary statements which did and did not meet HTA eligibility criteria. Only 8% of the summary statements were rated as significantly interesting; 100% of these were for common conditions and 71% had enough evidence to draw conclusions. 50% were significantly uninteresting. Reviews that lacked sufficient evidence to draw a conclusion were less likely to be significantly interesting (7% vs. 12%). These findings could then be used to inform future prioritisation processes, including for primary research.

#### Specific roles: refereeing and review readability

Wale (2010) identified a wide range of roles undertaken by consumers (in line with other evaluations) but the majority of CRGs (38/47) wanted to involve consumers in order to improve readability of reviews (38/47) and plain language summaries (36/47) [6]. Commenting on reviews and protocols was also the activity most commonly reported in both 2006 and 2009 surveys of consumers: 54% and 67% of consumers were engaged in these activities. Plain language summary preparation was much less commonly reported (25% in 2006 and 18% in 2009). Of those involving consumers just over half of groups felt that they were gaining the desired benefits, while 14/35 were unsure. In line with this, only around half of consumers felt that their involvement made a positive difference and many found it difficult to assess their impact.

The slightly earlier review of monitoring forms and reports submitted by CRGs (Zhang 2008) had found that fewer than half (23/51) involved consumers in the refereeing of all their protocols [8]. Of those reporting that this was not the case (27/51), 14 provided details stating that they planned to have universal consumer input in the coming years. Wale 2010 did not report on whether the number of CRGs with universal consumer input to reviews had increased in line with the importance placed upon it [6].

The organisation-wide emphasis on consumer involvement in peer refereeing was reflected in the focus of case studies from CRGS. The group with the most evaluations (four), and the most in-depth evaluations was the Pregnancy and Childbirth group. All of these focused on consumer involvement in peer-refereeing.

One of these studies involved a comparison of 2 models of consumer refereeing [15]. This compared one model where a consumer panel was used for the period up to 2007 with a model of peer review as commonly used for other reviews. The consumer panel model was highly acceptable to both consumers and editors and was discontinued for cost reasons. The peer referee model was less popular with both groups and was viewed by editors as producing less consistent, and less relevant feedback. The impact of the change was regarded as potentially cost-effective but the loss of consumer-led initiatives and absence of appropriate input to some reviews were regarded as negatives.

Previous evaluation of consumer input to this group from editors was positive, citing differences (not further specified) which the consumers made to final reviews. Review authors were more varied in their views while consumers were all uncertain as to the impact of their work due to lack of feedback on its quality and input into the final review (Horey 2005) [38].

Other reports on this group also dated from the period in which the consumer panel was active [16, 17, 39]. One examined the impact of consumer feedback on a specific topic-based subset of reviews and identified the specific types of useful feedback [39]. This was the only study which reported the relationship between consumer involvement and specific changes to reviews which resulted from the involvement. An audit of peer review over the first 2 years of the panel identified similar issues raised [16, 17]. Both studies identified positive impacts of consumer participation on the review process.

The Cochrane Skin Group published a case study which reported universal peer refereeing [19]. This group also reported increasing numbers of consumers and activity, and an almost unanimous view among consumers that their contribution was important and significant [18, 19].

Across the included studies which reported on peer refereeing, several types of contribution were identified. These addressed the relevance, conduct and accessibility of reviews. Key contributions included: providing and seeking clear rationales for reviews; ensuring the use of outcomes that matter to patients including adverse events/adverse effects of treatments; raising methodological concerns and queries about interpretation of data; seeking explanation of terms and making suggestions about accessibility, sensitivity, precision and clarity of language; and practical suggestions e.g. addressing potential conflicts of interest; amending titles.

Many of the reports identified ways in which the CRG felt that consumer involvement impacted on both reviews and the group more widely. These were not quantified and no report provided an audit trail for the impact of consumer input on the final review. Reports from three of the CRGs were uniformly positive but did not report specific examples of impact. Consumers in these groups clearly felt valued, however. Some in-depth studies on the Pregnancy and Childbirth Group were more equivocal. Whilst editors stated that input resulted in specific changes to reviews, review authors shared a range of views about the impact on the reviews, and consumers and consumer co-ordinators were uncertain about the impact of their contribution (Horey 2005, Gyte 2005) [38, 40]. Exploration of a subset of reviews, which identified positive contributions, supports the suggestion that this uncertainty may result from a lack of feedback (Horey 2004) [39].

The view that more investment in obtaining consumer feedback results in more valuable input was supported by Gyte’s 2011 comparison of two models for consumer comments on reviews, [15] and by the pilot approach of Skoetz (2005) who reported on the benefits of running a focus group to obtain input, although this was not specific to reviewing [23].

### Impacts of consumer involvement

Impacts of consumer involvement were characterised in different ways by the included studies. Some of these have been discussed in detail in consideration of individual roles for consumers. The cross-organisational study by Kreis identified only one formal evaluation of consumer involvement and this was the survey by Wale (2010) of Cochrane [6].

Several informal evaluations identified by Kreis’s survey were all positive about impacts on relevance and usefulness of reviews. Some identified concrete examples of differences made by consumer involvement; others reported that the benefits were less tangible. Indirect benefits to all involved parties (organisation, research groups and consumers) were also commented on.

Tangible beneficial impacts on review relevance and quality were documented by studies of Canadian and American organisations [10, 11]. In the Canadian HTA programme, impact assessments by both researchers and stakeholders was positive, identifying increasing depth, relevance and usefulness of the final review. Stakeholder involvement early in the review process was identified as resulting in broader and more inclusive literature searches and more clearly defined research questions. Later involvement was associated with identification of important issues around the clarity of the final report and the recommendations made. The US DERP noted that all of their reports underwent some basic editing changes made on the basis of the comments received. In several cases significant changes were made to methods sections. In many reviews a small number of additional studies were identified by the public; these were added to the reviews and constituted changes with the potential to alter the outcome of the review. Changes in inclusion criteria as a result of comments were rare but in one case they were broadened to include observational studies.

Most of the case examples of individual reviews were not Cochrane reviews and were highly variable in their level of involvement. The two Cochrane examples were reviews of physiotherapy for stroke (Pollock 2015) and of treatments for degenerative ataxias (Serrano-Aguilar 2009) [27, 28]. Both reported high levels of involvement throughout the review processes and substantive changes in the review process and the final review as a consequence of consumer involvement which was integral to the review.

The theme of indirect benefits was noted in the reporting of the CRG case studies, which highlighted that their teams were strengthened by consumers who were responsible for the inclusion of different perspectives; incorporation of quality improvement mechanisms; a more open and inclusive process; and more collaborative and stronger teams. It is notable that many of the activities where highly active CRGs reported consumer involvement – such as peer mentoring and partnership development – may themselves be characterised as impacts of involvement, in that they demonstrate the development of a highly skilled and motivated consumer group which is greatly empowered. In some cases the way in which we define an impact and an involvement activity should be considered fluid.

General impacts which were identified across studies included: increased relevance and timeliness; better dissemination including greater accessibility; and a reduction in bias. It seems reasonable to suppose that some of these benefits were derived, at least in part, from the improved processes identified by the impacts on the research groups.

The intangible nature of some of the benefits that were documented may explain some of the lack of certainty about impact which was documented in many of the studies, particularly relating to consumers’ views of their involvement in peer refereeing.

#### Barriers to consumer involvement

CRGs responding to Wale 2010’s survey and who did not have consumer involvement in their reviews, identified the need for more staff resources and guidance on consumer involvement as barriers to introducing it; a minority also identified the appropriateness of reviews for consumers as an issue [6]. Responses in Zhang (2008) identified the (lack of) relevance or interest of reviews to consumers, timescales of review groups and funding for the identification of consumers as barriers to universal consumer review [8]. Zhang 2004, which was the only study to focus on a non-Western country, identified a number of barriers to consumer involvement which applied in China [9]. These included language, information scarcity, cultural differences, education, funding, communication system difficulties and lack of facilities.

These difficulties may be present in other developing countries. Wale (2010) identified the geographical location of participating consumers as overwhelmingly in developed countries; two-thirds were from the UK and North America with others in Australia and Europe and only 4/63 from Asia or the Middle East [6]. This is supported by data from the CRG case studies which reported similar patterns, with variations for the location of the editorial bases (e.g. Canada, Germany) [20, 22, 23].

Barriers to consumer involvement identified by multiple organisations, including Cochrane, were lack of time and resources, uncertainty over how to identify the “right” consumers and concerns about possible negative impacts on scientific rigour [13]. Interestingly none of the case studies of CRGs focussed on barriers to involvement, although they did note some enablers; this may be a reflection of a local track record in overcoming such barriers.

## Discussion

### Summary of findings

The studies we identified had publication dates ranging from 1999 to the present. Most of the evidence identified by this review related to Cochrane, although some studies identified and evaluated other organisations’ involvement. In some instances, these covered the impact of novel strategies such as post-publication forums open to the general public.

Within Cochrane there were evaluations across the organisation as a whole and within individual review groups (CRGs). Evaluations and reports on individual CRGs related to only four of the CRGs, although many more CRGs were identified as having considerable consumer involvement by collaboration-wide surveys and audits.

The great majority of organisational level evidence related to Cochrane; however only a minority of the individual reviews where an identified publication documented or evaluated the impact of consumer involvement, related to Cochrane reviews. This may of course reflect the fact that consumer involvement in Cochrane reviews is less novel, more routine and less likely to be identified as something “different enough” to be worthy of a separate publication.

It was noteworthy that many of the identified case studies of individual reviews explicitly reported that consumer involvement led to changes in the review. It was therefore easier to be confident that tangible impacts of consumer involvement occurred than was the case with CRG or organisational level appraisals. This presentation of audit trails of consumer input and the subsequent changes to reviews was documented explicitly by only one CRG study, [39] and by none of the organisational studies. Given that these linkages were possible in all the reviews with substantive involvement, it would be worth considering methods for reporting consumer input, at a structural level, in future analyses.

### Emerging themes

Although Cochrane is an international organisation and its reviews are increasingly available in multiple languages, the focus of the identified research revealed overwhelmingly Anglophone involvement and heavy concentration of consumer involvement in high income countries. There was limited representation in published studies from low and middle-income countries and countries where English is not a main language, with only two studies focused on countries outside these categories (Germany and China) [9, 23].

One theme, which emerged from the analysis, is the increasing level of consumer involvement in terms of the number of CRGs which routinely involve consumers in some way in some or all of their reviews. Consumers are identified as contributing throughout the research cycle, from priority setting; outcomes identification; commenting on protocols reviews and plain language summaries; helping to disseminate knowledge; and playing important roles within Review Group teams. However the involvement of consumers often remains focused on providing input as consumer referees to reviews or protocols and in assisting with the provision of plain language summaries. Involvement of consumers in other more “upstream” activities such as prioritisation of topics and outcome identification does not appear to show the same pattern of increasing activity. In general there is evidence of inconsistency across the Cochrane network, in terms of commitment to involvement, resources directed at involvement, and the variety of different approaches taken.

Some individual Review Groups are well-represented in the literature revealing a commitment both to involving consumers and to publishing the results of their work (for example the Cochrane Muscular-Skeletal Group and the Cochrane Pregnancy and Childbirth Group) [15, 20, 22, 39–42]. However, in general, there is a scarcity of information about the extent and nature of consumer involvement in Review Groups and a rarity of audit of involvement processes such that the impact of activity is unrecorded in published literature.

A range of perceived barriers to effective involvement across both Cochrane and other organisations are identified in the literature. These include a lack of resources, guidance, language and the difficulty of recruiting suitable consumers. A clear range of benefits from the involvement process are also identified including the use of consumer-oriented and more relevant outcomes, changes in language, informing of methodology and adding “depth” to the review. Whilst these perceived barriers and benefits are subjectively identified there is a shortage of objective evidence about what works.

A particularly notable finding was that, at an organisational level, consumers themselves are often uncertain about the value of their contributions; editors and authors appear to be more confident in this than those who are providing the consumer input [6, 10]. This finding did not appear to be reflected in some of the reports from CRGs and external organisations with high levels of commitment to consumer involvement such as the Pregnancy and Childbirth CRG [15, 38–42]. It may be that improved communication between consumers and the wider review team may make the impact of their involvement clearer to consumers.

One theme which did clearly emerge from the studies of individual CRG processes was that the level of benefit derived from consumer involvement was often proportional to the level of investment of time and resources in obtaining and facilitating that involvement. Structures and processes which were designed to support meaningful input from consumers resulted in more engaged consumers and, ultimately, higher value consumer contributions [6, 15].

### Limitations of this review

This work does not represent a full systematic review of the literature. Although we were able to have two authors independently screen the identified records from the databases (while blind to each other’s decisions), only one author assessed the abstracts from the Cochrane Colloquia. We were unable to obtain two records despite an international inter-library loan request. Both of these were in German and may have served to partially correct for the focus of the identified literature on involvement from English speaking countries. We were also unable to conduct a useful quality assessment of the included studies as they represented a diversity of study designs and approaches; many were also reported in abstract form only.

### Limitations of the included studies

Almost all of the included studies were case studies, case series or reports of surveys or audits. Only one adopted a comparative design and this took the form of a historical control. Many of the studies did not attempt to assess the impact of particular types of involvement and most of those which did supplied little or no evidence to support the assertions of impact. Although attempts to audit and describe processes have value they do not allow us to infer that reported outcomes are a consequence of these processes.

## Conclusions

This scoping exercise has identified evidence of highly variable levels and types of consumer involvement within and beyond Cochrane, but limited evidence for the impact of most methods and levels of involving people. Promising initiatives exist and where there is evidence of impact at the level of individual reviews and review groups it supports the need for properly resourced and supported processes in order to derive the greatest benefit from the lived experiences of consumers who are willing to be involved. Further, more rigorous evaluations are needed in order to determine the optimum approach to achieving this.

### Recommendations for further research and practice

Whilst there are considerable difficulties in conducting controlled studies in this field it would not be impossible to design a study which adopted a more rigorous approach to evaluating which models of organisational consumer involvement have greatest impact on the quality and relevance of the output. Different approaches to this might be considered.

In the interim CRGs should be encouraged to record and publish their experiences of consumer involvement, including any innovative processes they adopt. This would enable widespread and rapid adoption/exploration of processes which are found to be effective by particular groups. In particular CRGs should explore and document methods of involving consumers throughout the research cycle including prioritisation, outcome identification and dissemination to other consumers and consumer organisations.

Inclusivity and representativeness should be pursued by working towards ensuring consumers reflect, as far as possible, the population as a whole in respect of gender, age, ethnicity, disability, sexual orientation, socio-economic group, spoken language, developed/developing world. This might be achieved through targeted recruitment programmes to improve representation of currently under-represented consumers from low-income and non-English speaking countries.

## Additional files

Additional file 1: Table S1. Surveys at organisational level. **Table S2**: Surveys and case reports of involvement by individual CRGs. **Table 3**: Reports of involvement in individual systematic reviews - case reports. (DOCX 57 kb)

Additional file 2: Appendix 1. List of excluded or unobtainable studies. (DOCX 15 kb)

## Abbreviations

AHRQ: Agency for Heathcare Research and Quality
CCNet: Cochrane Consumer Network
CRG: Cochrane review group
DERP: Drug Effectiveness Review Project
EPC: Evidence-based Practice Centre
HTA: Health Technology Assessment
RCT: randomised controlled trial
UK: United Kingdom
US: United States of America
