# Supplementary material for: A systematic scoping review of the evidence for consumer involvement in organisations undertaking systematic reviews: focus on Cochrane
Source: Res Involv Engagem. 2016 Dec 21;2:36. doi: 10.1186/s40900-016-0049-4 (PMC5831869; doi:10.1186/s40900-016-0049-4)
Supplement: Supplementary file 1 — Surveys at organisational level. Table S2: Surveys and case reports of involvement by individual CRGs. Table 3: Reports of involvement in individual systematic reviews - case reports. (DOCX 57 kb) [file 40900_2016_49_MOESM1_ESM.docx]

**Supplementary Table 1: Surveys at organisational level**

| Study | **Methodology** | **Consumers** | **Results** | Impact |
| --- | --- | --- | --- | --- |
| **Cochrane** | | | | |
| Ghersi 1999, 2002 (3) | Development of a plan for consumer involvement in 4 CRGs  Audit of all consumer activities & impact of consumer refereeing | Only reported for Cochrane breast cancer group: 2 consumers on editorial committee involved in priority setting; 7 involved in design/conduct of reviews. | **Activity undertaken included**:   - Membership of editorial teams - Peer-review (more than 40 consumers) - Undertaking reviews - Producing "lay summaries" - Collaboration with external consumer organisations to publish synopses; - Publication of synopses /other CRG documentation in languages other than English. - Removal of unfriendly jargon from synopses & other documentation. - Coordination of workshops, seminars and presentations on CRG activities during consumer organisation conferences. - Trial identification (In breast cancer group) | Refereeing of protocols and reviews in the breast cancer group s resulted in important changes e.g. use of consumer-oriented outcomes and improvements in language. |
| Horey 2010 (7) | Audit using Archie and other methods of consumer activities and types of consumer involvement | Consumers registered on Archie in any capacity | **Identified consumers**   - 521 individuals registered as consumer reviewers (consumer referees) - 9 as consumer coordinators - 107 as handsearchers. - 723 active members of the CCNet. - Most (61%) consumer reviewers in the Cochrane were not members of the CCNet   **Consumer Roles**  Three types of consumers were identified:   - Consumer volunteers whose input is based on lived experience - Consumer facilitators who work to promote/support involvement; this may be funded or voluntary. Lived experience is supplemented by additional roles; not all people in these roles identify as consumers - Consumer organisation partners working as representatives or in liaison roles with consumer organisations | Three options identified:   - Current approach including the provision of project funding to the CCNet to support consumer volunteers; - Extending the remit of the CCNet to assume support responsibility for all consumers, including consumer facilitators and consumer organisation partners; - Establishing an administrative support unit to support CCNet, other volunteers, consumer facilitators and consumer organisation partners. |
| Kelson 1999 (4) | Survey of all types of consumer involvement & CRG perceived importance of consumer involvement  Focus on patient- derived outcomes | Consumers involved with CRGs | **Response**  35/42 CRGs responded ; 33 questionnaires were completed  **Overall involvement**   - 10 CRGs had no consumer members of which 4 were actively/intending to recruit.. - Others had between 1-10 (mean 3.6).   **Acceptance**   - 4 committed in practice (12%), - 22 committed in theory (66%) - 6 unconvinced (18%)   **Patient-defined outcomes activity**   - 19 had discussed patient-defined outcomes (58%). - 5 had done a literature search (15%) - 3 had produced a bibliography/ summary / review ( 9%) - 5 had incorporated literature into wider reviews (15%). - 12 had liaised with consumers/ consumer organisations (36%) - 2 had identified organisations to contact (6%). | - 8/12 rated the importance of consumer contributions; 6 reported a major role and 2 hoped that role would develop in future - Areas identified included informing methodology & reporting of reviews, participation in working groups, suggesting outcomes and priority setting. |
| Nasser 2013 (5) | Survey of groups of Cochrane review authors (groups/fields/networks)  Focus on use and nature of priority setting | All types of consumers involved in priority setting | **Response**   - 52/66 (78%) responded. - 29 (56%) had a process to inform prioritisation/selection of review topics   **Types of processes**   - Editorial processes where editors were asked for priority topics - Survey of consumers, reviewers, and editors of the review group - Comparisons of topics of finished Cochrane reviews with trials in the group trial register or recommendations from initiatives like the Database of Uncertainties about the Effects of Treatments (DUETs) - Web-based topic suggestion interface forms.   **Structured processes**   - 13 unique structured transparent processes in 15 Cochrane entities - All except 2 of the prioritization processes were to identify new topics for Cochrane reviews. - Some such as the James Lind Alliance (Incontinence Group) or the equity-oriented process of the Musculoskeletal group had wider additional aims.   **Stakeholder involvement**   - 2 processes involved 4 or more types of stakeholders. - 12 groups involved 3 types of stakeholders (researchers, practitioners, and patients) - 11 groups involved only 2 (researchers and practitioners in 10 cases, researchers and policy makers in 1). - 2 groups involved no external stakeholders. - Only 1 group involved the public and the press in their process, through the use of an online survey - Only 1/13 processes had a formal appeal mechanism for the process of prioritization. | - 2 Cochrane entities have developed structured strategies to evaluate the priority-setting process. - One also intended to provide potential recommendations for changes in strategic direction or resource allocation of its affiliated CRG. - One planned to share the final identified uncertainties with the UK DUETS, another planned to develop a database of priority reviews and evaluation of plain language summaries of priority reviews. |
| Wale 2010 (6) | Surveys of consumers (2006, 2009) and CRGs (2009) | Consumers listed as involved with CRGs | **CRG consumer involvement 2009**   - 47/52 CRGs responded - 35 CRGs involve consumers - 8 CRGs do not involve consumers - 4 CRGs did not answer - 38 CRGs wanted to involve consumers to improve the readability and/or quality of reviews - 36 wanted consumers to improve usefulness of plain language summaries. - 27 said inappropriateness of reviews was not a barrier to consumer involvement; 7 that it was - CRGs without consumer involvement identified need for more staff resources and advice/guidance as barriers   **Consumer activities 2006 (N = 63)**   - Commenting on reviews and protocols: 34 - Talking to others about evidence-based healthcare 29 - Plain language summary preparation 16 - Giving workshops 16 - Review author 11 - Handsearching trials 7 - Advisory group/editorial team 7 - Topic prioritisation 5   **Consumer activities 2009 (N = 66)**   - Commenting on published reviews 44/ 30 CRGs - Commenting on published protocols 34/ 25 CRGs - Raising awareness of evidence-based healthcare 26 /9 CRGs - Recruiting others 18/ 10 CRGs - Dissemination 17 /9 CRGs - Plain language summaries 12 - Co-author on review 10 | **CRGs 2009**   - Just over half of CRGs felt that they were gaining desired benefits of consumer involvement, 14 were not sure. - Consumers can have a negative impact by being overly critical.   **Consumers 2006/2009**   - Consumers find it difficult to assess their impact because they often do not receive feedback. - Half felt that their involvement makes a positive difference.   **Role of CCNet**   - 38 CRGs felt CCNet was important, 7 had no view, 2 did not answer - Consumers were less sure - 20 CRGs had referred consumers to CCNet guidance; 18 did not know - Most CRGs did not feel up to date with CCNet activity |
| Zhang 2004 (9) | Case study of new involvement in China  Audit of activity and barriers to involvement | Group of professional consumers including medical and English students, hand searchers, editors, public health researchers, librarians and active patients | **Barriers to involvement**   - Language - Information scarcity - Cultural differences, - Education, - Funding - Communication system, Lack of facilities. - These were quite different from those in developed countries. | **Activities of new group**   - Obtained information - Translate and rewrite the consumer synopses in plain Chinese language (every issue) - Provide consumer comments for review groups (provided 14 protocol comments), - Identify the needs for non-professional consumer involvement |
| Zhang 2008 (8) | Review of monitoring forms and reports submitted by 51 CRGs | Consumer involvement in all protocols and reviews | **Reported consumer involvement in all protocols/reviews**  23 CRG answered ‘yes’, 27 CRGs answered ‘no’, 1 CRG did not respond.  **CRGs responding “yes”**   - 18 CRGs provided details including strategies to encourage authors to have consumer input - These included: assisting authors when they found difficulties; listing potential consumers and trying to match their areas of interest to topics; identifying consumer coordinators or involving consumer as co-authors; providing training for consumers; establishing links with CCNet   **CRGs responding “no”**   - 18 CRGs provided details of which 14 stated they would plan to have consumer input in the coming years. - Barriers were relevance/interest of Cochrane topics/protocols/ reviews to consumers; timescale for review groups; lacking funding to identify consumers | **No impacts were reported** |
| **Non-Cochrane organisations** | | | | |
| Keown 2008 (10) | Retrospective case series of 22 reviews in Canadian HTA-style organisation  Audit of involvement opportunities across review processes for consumer involvement &  impact of consumer involvement | 80 stakeholders involved over 4 years | **Opportunities identified**   - Stakeholder topic consultation (research question); - Stakeholder input meeting (literature search and inclusion decisions); - Stakeholder review team member (throughout review process); - Stakeholder reaction meeting (drafting and final report production) - Involvement in dissemination. | - Review teams felt stakeholders’ input added depth to the review. - Timing of stakeholder participation leads to specific advantages. Early involvement resulted in broader and more inclusive literature searches and more clearly defined research questions. - Later involvement identified important issues relating to the clarity of the final report and the recommendations. - Stakeholders appreciated being included. They feel researchers can learn from their “frontline” experience and that including their point of view will make systematic review findings more useful and relevant. Involvement increased interest in communicating review findings to colleagues |
| McDonagh 2006 (11) | Analysis of volume and type of changes made to 26 drug class reviews published on the website of the US Drug Effectiveness Review Project (DERP) as a result of consumer comments | Members of the public | **Comments received**   - All 26 reports received comments through the website. - Volume of comments varied by report topic - Reviews of drugs for psychiatric indications received the highest volume. | - Basic editing changes were made on all reports based on the comments. - Several significant changes were made to methods sections. - Changes with potential to alter the outcome were due to additional studies added to the review; for many reviews a small number of additional studies were identified by public comments and added to the review. - Alterations in inclusions criteria were extremely rare, but in one case they were broadened to include observational studies |
| Vale 2012a (12) | Semi-structured questionnaire to researchers at the UK MRC clinical trials unit assessing level of consumer involvement in research including systematic reviews | All types of consumers involved in the MRC work | **Response**   - 138 completed questionnaires (86%). - Studies conducted from 1989 onwards - 50% were in cancer; 30% in HIV and 20% were in other disease areas including arthritis, tuberculosis and blood transfusion medicine.   **Involvement (all)**   - 43 studies (31%) had some consumer involvement - 34 of these were RCTs, most commonly as members of trial management groups (TMG) (88%)   **Involvement (reviews)**   - 23 (17%) were systematic reviews (number with involvement NR) - 1 on prion disease was highlighted as example of useful involvement, 1 in oncology (Vale 2012b) as an example of further research resulting | - Most researchers identified benefits of involving consumers in studies - Most studies did not use consumer involvement |
| **Multiple organisations** | | | | |
| Bastian 2011(14) | Web-based survey of views on level of interest of Cochrane review summary statements of reviews which did/did not meet HTA eligibility criteria | Employees &  Mmembers of patient information committee  CCN email list/international evidence-based medicine list | **Response**   - 194 completed surveys obtained (response rate NR) - 95 (49%) of surveys from Germany; 45% identified as health professionals; 60% female   **Findings**   - 8% of summary statements were rated significantly interesting; 50% were significantly uninteresting - Reviews lacking sufficient evidence to draw a conclusion were less likely to be significantly interesting (7% vs. 12%). - 100% of significantly interesting judgements were for common conditions; 71% had enough evidence, - An uncommon condition and not enough evidence for a conclusion were predictive of being significantly uninteresting (93% of 15 reviews). | **No impacts were reported** |
| Kreis 2013 (13) | In-depth, semi-structured interviews with key informants and review of organizations’ websites for 17 US-based and international organisations involved in commissioning or conducting systematic reviews.  Opportunities for and impact of consumer involvement | Different types of consumers (individual patients, consumer advocates, families and caregivers), recruited mainly through patient organizations and consumer networks. | **Involvement**   - Organisations included federal agencies, payer and provider organizations, private and university-based organizations, the Cochrane and Campbell Collaborations and 2 CRGs (musculoskeletal and pregnancy and childbirth). - 7/17 organizations usually involve consumers. - These were Cochrane, the Campbell Collaboration, 2 CRGs, the AHRQ, Oregon Evidence-based Practice Centre (EPC), Johns Hopkins EPC. - Other organisations occasionally involve consumers in reviews or involve them regularly in other processes   **Type of involvement**   - Topic suggestion - Input on key review questions - Comments on draft protocols and reports, co-authors - Sit on an advisory group. - Extensive involvement in priority setting was reported by AHRQ and its EPCs - Some offer research methods training; 1 provides guidance on consumer involvement   **Reasons for not involving consumers**   - Lack of time/resources, - Possible negative impact on scientific rigour - Unsure how to find right consumers | **Informal evaluation**   - All key informants were positive about the impact of consumer involvement, identifying the potential beneficial effect on the relevance /usefulness of reviews. - Some reported concrete cases where the involvement of consumers had made a difference, others that benefits are not always easily tangible - Indirect benefits were identified at the level of the consumer, research group and organisation   **Formal evaluation**   - The only formal evaluation identified was the CCNet 2009/2010 review (Wale 2010). |

**Supplementary Table 2: Surveys and case reports of involvement by individual CRGs**

| **Study** | **Methodology** | **Results** | | **Impact** |
| --- | --- | --- | --- | --- |
| **Pregnancy and Childbirth Group** | | | | |
| Grant-Pearce 2003/2004; Horey 2005,Gyte 2005 (37, 39-41) | Semi-structured interviews with 18 members including editors (4), reviewer authors (5), consumers (5) and consumer coordinators (1). These were representatives of 300 individuals involved in group which has a consumer panel which includes 72 volunteer consumers who choose which reviews/protocols they wish to comment on and 3 consumer co-ordinators | **Quality of consumer input**   - Reviewers and editorial staff especially varied in judgement of the quality of consumer input providing personal views on: objectivity, relevance, conciseness, coherence, constructive tone - Highlighted responses were generally positive - Some concerns expressed about length of responses and possible “agendas”   **Consumer suggestions for improvement**   - Opportunities for training: including explanation of the consumer role and its effect, and skill development in critical appraisal; - Improved communication and feedback: such as regular newsletters, opportunities to discuss issues with other consumers, and copies of the published protocol and/or review;. - Targeted information and support, including individualised induction packs and access to mentors. | **All groups**   - Consumers brought added value in the form of a different perspective. - This encouraged methodological clarity;   use of plain English; identification of different and more meaningful outcomes; more comprehensive enquires   - Consumer input improved the final review but earlier consumer input may be beneficial   **Editors**   - Consumer input did make a difference to final reviews in specific ways   **Reviewers**   - Responses ranged from ‘yes absolutely’ to no‘ the overall message or results did not change’   **Consumers**   - All consumers responded with uncertainty, citing lack of feedback on the quality of their input and its position in the final review.   **Consumer co-ordinators**   - Generally felt it did make a difference, though there was uncertainty as to contributions within the final product | |
| Gyte 2011 (15) | Survey (interviews) of editors (7/7 responded) and consumers (7/14 responded)  Comparison of 2 methods of consumer referee/feedback provision  Consumer panel (1998-2007) all consumers in panel invited to comment on all protocols and reviews.  Peer review process as used for other referees (2007-2011) (73 consumers | **Panel**:   - Highly acceptable to both consumers and editors - Not feasible over longer term as dependent on funded coordinator position   **Consumer peer review:**   - Qualified acceptability among consumers and editors. - Most wanted return to other model. Several consumers no longer participating.   **Editors**   - Feedback is more variable or missing. - “outcomes that matter” are no longer highlighted. - More work of editors and authors as comments may be lengthy and/or not directly relevant.   **Consumer s:**   - Possible reduction of author burden with less feedback - Restriction of consumer choice on what to comment on and may diminish evidence-based value of response | **Panel**   - Effective consumer input; both useful to review content and the summary helpful to authors and editors   **Consumer peer review**   - Consumer input sometimes difficult to integrate. - Possibly cost effective but input sometimes absent and loss of consumer-led initiatives. | |
| Horey 2004 (38) | Analysis of feedback from the consumer panel on protocols/reviews on hypertension in pregnancy and caesarean deliver (data not presented) | - Consumer feedback raised methodological concerns, queried interpretation of data, sought explanation of terms and rationale, made suggestions about potential outcomes of interest, made practical suggestions e.g. how to address potential conflict of interest and how titles could be amended. - Consumers identified topics of interest to consumers, such as, side-effects of treatment and potential adverse effects. | Consumer feedback in the review process relating to hypertension in pregnancy contributed positively to the review process. | |
| Sakala 2001a,b (16, 17) | Audit of peer review by consumer panel over 2 years | - More than 50 people participated in the panel - 46 protocols and 40 reviews have been evaluated by the group with others in progress. Numbers evaluating protocols ranged from 1-7. - Common concerns identified included: language (accessibility, sensitivity, precision, clarity); clear rationales for reviews; priority setting issues; types of outcomes assessed (relevance to patients and adverse effects). | - Expanded the team and strengthened collaboration - Helped reduce bias and increase relevance/ accessibility, - Incorporated quality improvement mechanisms and created a more open and inclusive process. | |
| **Skin** | | | | |
| Collier 2005 (18) | Survey of 19 Cochrane Skin Group consumers active in countries other than UK/US (NR if this is total N) | - Consumers were drawn to health topics relevant to the health of the consumer, a family member, or a friend. - Areas needing improvement identified: communication between CSG members; education and training experiences for consumers; decreasing the use of medical jargon; increasing awareness of CSG consumers in prominent medical organizations such as the American Academy of Dermatology and the American Medical Association. | Consumers expressed an almost unanimous belief that their contribution was important and significant | |
| Reddi 2013 (19) | Descriptive analysis of peer review and authorship | - About 100 active consumers are members of the CSG and are involved at many levels. - A relevant consumer is always involved in the peer-review process and often acts as a co-author. - Initially, the National Eczema Society and the Vitiligo Society of the UK provided the majority of consumers. Now many other patient advocates are involved. | No impact reported | |
| **Musculoskeletal** | | | | |
| Rader 2011 (21) | Case study of roles for consumers | **Identified specific roles included**   - Participating in priority-setting exercises to help choose topics - Commenting on and peer-reviewing systematic reviews and protocols, - Developing plain language summaries and decision aids for patients - Promoting systematic reviews to local contacts | **New consumer-driven initiatives**   - Peer mentoring program - Peer recruitment - Consumers joining author teams - Consumer participation in other knowledge translation activities.   **Overall impact**  Consumer partner involvement enriches musculoskeletal systematic reviews and helps increase their relevance | |
| Shea 1998 (22) | Report on setting up and initial activity of consumer group | **Activities over 2 years included:**   - Providing advice on consumer needs, eg. drug-to-drug comparisons instead of placebo comparisons; - Producing 11 plain language summaries; - Providing contributions to the newsletter; - Assistance developing partnerships with other like-minded organizations - Assistance seeking funds. | - Involvement of consumer group has led to new consumer-driven initiatives. - Having consumers involved as partners in the review group process results in more research truly relevant to consumer and clinician concerns | |
| Shea 2005 (20) | Case study of consumer involvement | **Activity of consumers**   - Setting research priorities - Production of reviews (editing/writing) - Promoting awareness of reviews including conference participation - Recruitment - Training - Development of consumer-friendly formats, - Production of consumer summaries and newsletter reports, - Input into knowledge translation and funding bids. - Provision of training to consumers. | - Priority setting involvement of consumers means the CRG is more confident that their reviews are timely and relevant to consumers. - Consumer involvement in the peer review process makes reviews more likely to answer the questions consumers have. - Consumer members help the CRG actively ensure that the information from their systematic reviews reaches consumers, and that this information is in a usable format for use in evidence-based decision making. | |
| **Haematological malignancies** | | | | |
| Skoetz 2005 (23) | Case study of a pilot project to build a consumer network; Report on tailored workshops held in Germany | - 17 people attended the basic workshop; 8 attended the advanced one - All participants found the research experience of practical benefit to them; they were generally positive about their experiences. - Initially there were two international consumers actively involved; this increased to 6 along with 10 German volunteers. - 3 patients educated in the workshops are active in the Cochrane Breast Cancer and Cochrane Colorectal Cancer group. - In 15 months eight protocols and three reviews were refereed, and the consumer synopsis of four existing reviews were translated into German | **Consumer views**  Following the workshops there was an increase in positive views among consumers, who felt that the concerns of patients were extremely influential in the review process.  **CRG view**  The best and most comprehensive consumer feedback was from a focus group on a review; consulting, listening and discussing were more valuable than just sending out forms. | |

**Supplementary Table 3: Reports of involvement in individual systematic reviews - case reports**

| Study | Topic | Consumers | Methods | Results | Impact |
| --- | --- | --- | --- | --- | --- |
| Braye 2005 (24) | Law in social work education | 15 participants with social services user or carer experience | Stakeholder conferences | Consumers contributed to finalising the research question (1^st^ conference)  Consumers evaluated data, reviewed emerging results, made recommendations for the report and looked at the wider implications of the findings (2^nd^ conference) | - The involvement of consumers helped to ensure the research was fit for purpose and its outcomes credible. - Methodological rigour was enhanced and the likelihood of results being valid and useful increased. |
| Rees 2007 (33) | MSM | Representatives of organisations relevant to MSM | The advisory group was informed by a systematic map of the literature and then used formal consensus development methods to identify priorities for the research. They then commented on initial findings and communicating the results as well as the draft report | The advisory group advised against focusing on the concept of risk sexual behaviours and in favour of focusing on the concept of gaining control of one’s own health. An important threshold date was recommended and adopted (1996 when HAART became widely available). Important subgroups of vulnerable men were identified. An additional outcome was identified and became the most emphasised in the review | The involvement of consumers shaped the scope and focus of the review throughout the process and at a number of levels. |
| Carr 2007 (42) | ECT | 2 authors with direct ECT experience plus additional reference group members representing user/voluntary groups | Review authorship (including lead investigator) and reference group meetings | Advice on location of literature including identification of important unpublished study  Advice on methodological limitations of included studies and the heterogeneity between them  Recommended including emotional impact of ECT in review outcomes. | - Findings were changed as a result of consumer input into the review process with persistent memory impairment and issues around provision of sufficient information for informed consent being identified. - This impacted NICE guidance. |
| Stewart 2007 (32) | Newborn screening | 22 parents whose babies had been screened (representing range of screening results) | Expert groups at each stage and consulted and piloted resulting policies and resources; this was development of methods based on prior work including systematic reviews as well as review which formed part of the project | Difficult to determine the extent to which the objectives ere achieved | Not reported |
| Vale 2012a (30) | Prion disease | NR | NR | Questioning of results and processes was helpful | Help preparing lay summary and disseminating results to wide audience were appreciated |
| Oliver 2015 (35) | Childhood obesity | Groups of young people aged 12-17 years | Two consultative workshops one on views about obesity (review 1) and one on links between obesity and educational attainment (review 2) | Young people advised on the robustness of the review synthesis and helped develop the review implications (review 1). They helped identify plausible links mediating between obesity and attainment and helped develop review implications (review 2) | - Researchers had greater confidence in review findings after checking that themes identified by consumers as important were appropriately emphasised. - Limitations in the scope of primary research were also identified. |
| Campbell 2013; Pollock 2015 (26, 27) | Physiotherapy approaches for patients with stroke | 13 purposively selected stroke survivors, carers and physiotherapists. | Stakeholder group  Nominal group techniques were used to reach consensus decisions around review aims and methods, focusing on clinical relevance. Votes on key issues. Ongoing contact maintained after 3^rd^ meeting | 84% of group members disagreed with current categorisation of interventions. 100% agreed with inclusion of international trials. Current intervention categories should be amended to enable inclusion of all international evidence  There are limitations with current physiotherapy taxonomies and concerns surrounding the relevance to clinical practice in the UK  Group members reached consensus over 27 clearly defined treatment components, which were to be used to categorise interventions within the review (12/12 agreed or strongly agreed), and at meeting 3, they agreed on the key messages emerging from the completed review | - The scope and format of the review were changed to reflect consumer input - 9/13 members completed an evaluation form at the end of meeting 3, all respondents strongly agreed that the views of the group impacted on the review update, that the review benefitted from the involvement of the stakeholder group and that they believed other Cochrane reviews would benefit from the involvement of similar stakeholder groups. Members felt their opinions were valued. |
| Jamal 2011 (25) | Public health: school environment interventions for children and young people’s health | A pre-existing group of young people brought together to advise on public health research | Face-to-face to consultation methods, supplemented with online social networking  Views were sought at three stages: at the beginning for general views; half-way through for views on the map of identified evidence; near the end when review results were presented and discussed | The first and second consultations helped researchers to understand the policy problem in its current context and informed development of inclusion criteria for priority review areas.  Face-to-face consultation was better at engaging young people but social networking gave the review team greater flexibility to elicit further views when unanticipated issues arose that needed immediate input. | Consulting with young people alongside other stakeholders added considerable value to the review, especially in terms of helping the review team to prioritise in a relevant and meaningful way. |
| Oliver 2001 (34) | Public health: smoking cessation in pregnancy | Mothers who are current or former smokers | Antenatal smoking cessation programmes and their evaluation were discussed with women and health promotion specialists, prior and subsequent to the review | Consumer concerns were addressed in the methods by abstracting data systematically about process measures with a broad range of maternal, foetal and infant outcome measures | - Missing outcomes of importance to women identified (not measured in past trials. This has been partially redressed in a trial that included breastfeeding and maternal mental health as outcomes. - Information important for the uptake of recommendations was revealed about the content, delivery and acceptability of interventions. |
| Serrano-Aguilar 2008. 2009 (28, 29) | Neurology: treatment for degenerative ataxias | 53 patients with degenerative ataxias from different regions of Spain (100% of those invited) | Involvement of patients using the Delphi method, with three rounds via e-mail. The first round obtained information on treatments used and relevant self-perceived health problems. The following 2rounds were used to prioritize and achieve consensus on answers | The participation rate was 100% for all rounds. The most relevant self-perceived health problems were limitations in activities of daily living, visual and auditory problems and diminished self-esteem. The bibliographic search for the systematic review was enriched by these patient contributions. | - No study offered information on treatment effectiveness for the following problems prioritized by patients: ADL, social relationships, disease acceptance and quality of life. - Effective participation of patients can extend the value of systematic reviews to ensure they respond to both clinicians’ information needs and patients’ expectations. |
| Smith 2009 (31) | Nursing midwifery & social work | Members from 24 national consumer organisations | Developing a service user reference group | Invitations to participate produce mixed responses. Pragmatism informed the course of events as well as planning. The representation framework helped to judge success, especially useful for differentiating between service users and those from organisations which represent them (and are not themselves service users)  The right environment is conducive to involvement and this crucially includes preparation  Decisions made at the early conceptual level of research design affect service users and researchers in complex ways. | Involving service users helped us to access information, locate the findings in issues that are important to service users and to disseminate findings |
| Vale 2012b (30) | Oncology: chemoradiation for cervical cancer | Six women, each of whom had received treatments for cervical cancer | Patient research partners took part in a variety of activities around the systematic review  3 also became involved in a related research project and also participated in the evaluation of the process and outcome. | Researchers and patient research partners felt that they had learned a lot from the process and considered it to have been a positive experience.  Consumers added a different viewpoint and both provided and gained insights  The researchers felt that because of resource implications, patient involvement in future systematic reviews would probably have to be prioritized to those in which the greatest impacts could be achieved | Consumer involvement directly led the researchers to another research project with a greater focus on late side effects of treatment. It also motivated them to publish an editorial with the partners, discussing the concerns of women after treatment for cervical cancer |
