# Supplementary material for: A systematic scoping review of the evidence for consumer involvement in organisations undertaking systematic reviews: focus on Cochrane
Source: Res Involv Engagem. 2016 Dec 21;2:36. doi: 10.1186/s40900-016-0049-4 (PMC5831869; doi:10.1186/s40900-016-0049-4)
Supplement: Supplementary file 2 — List of excluded or unobtainable studies. (DOCX 15 kb) [file 40900_2016_49_MOESM2_ESM.docx]

**Appendix 1: List of excluded or unobtainable studies**

**Excluded studies**

Barber R, Boote J D, Cooper C L. Involving consumers successfully in NHS research: a national survey. 2007; Health Expectations 10(4):380-91.

Bastian H. Consumer refereeing: what are the best ways to do it? 1998;(4):9-11.

Boote J, Wong R, Booth A. 'Talking the talk or walking the walk?' A bibliometric review of the literature on public involvement in health research published between 1995 and 2009. 2015; Health Expectations 18(1):44-57.

Brett J, Staniszewska S, Mockford C, Herron-Marx S, Hughes J, Tysall C, et al. Mapping the impact of patient and public involvement on health and social care research: a systematic review. 2014; Health Expectations 17(5):637-50.

Chalmers I. The Cochrane Collaboration: preparing, maintaining, and disseminating systematic reviews of the effects of health care. 1993; Ann N Y Acad Sci  703:156-63; discussion 163.

Fleurence R, Selby J V, Odom-Walker K, Hunt G, Meltzer D, Slutsky J R, et al. How the patient-centered outcomes research institute is engaging patients and others in shaping its research agenda. 2013; Health Affairs 32(2):393-400.

Forsythe LP, Szydlowski V, Murad MH, Ip S, Wang Z, Elraiyah TA, et al. A systematic review of approaches for engaging patients for research on rare diseases. 2014; Journal of General Internal Medicine 29(SUPPL. 3):S788-800.

Gold R, Whitlock E P, Patnode C D, McGinnis P S, Buckley D I, Morris C. Prioritizing research needs based on a systematic evidence review: a pilot process for engaging stakeholders. 2013; Health Expectations 16(4):338-50.

Gooberman-Hill R, Burston A, Lenguerrand E, Clark E, Johnson E. Patient involvement in clinical research: A qualitative evaluation of impact. 2013; Trials14:39DUMMY-.

Grande S W, Faber M J, Durand M A, Thompson R, Elwyn G. A classification model of patient engagement methods and assessment of their feasibility in real-world settings. 2014; patient Education and Counseling 95(2):281-7.

Hermans D, Price J. Consumer involvement in Cochrane reviews: is it worth the trouble? [abstract]. 2001;8-.

Kelly P, Broughton H, Coghlan R, Davies J, Davies N, Jadad A, et al. Consumer representation on the Cochrane Musculoskeletal Review Group. A systematic approach. 1998; Cochrane Consumer Network Newsletter, 5:10-1.

Liberati A. Consumer participation in research and health care. Making it a reality. 1997; British Medical Journal 315(7107):499-.

Murad MH, Prutsky G, Elraiyah T, Wang Z, Nabhan M, Shippee N, et al. Patient engagement in research: a systematic review. 2014; BMC Health Services Research 14, 89-.

Nasser M, Fedorowicz Z, Souza R F, Sharif M O, Hu N, Sequeira P, et al. Evaluating three informal capacity building networks for systematic reviews. Poster presentation at the Joint Cochrane and Campbell Colloquium; 2010 Oct 18-22; Keystone, Colorado, USA [abstract]. 2010;Suppl(Cd000002):94-.

O'Connell D, Mosconi P. An active role for patients in clinical research? 2006; Drug Development Research 67(3):188-92.

Oliver S, Selai C, Oakley A. Consumers as peer reviewers of systematic reviews of effectiveness [abstract]. 2001;47-.

Prictor M, Hill S. Cochrane consumers and communication review group: Leading the field on health communication evidence. 2013; Journal of Evidence-Based Medicine 6(4):216-20.

Rader T, Pardo Pardo J, Stacey D, Ghogomu E, Maxwell L J, Welch V A, et al. Update of strategies to translate evidence from cochrane musculoskeletal group systematic reviews for use by various audiences. 2014; Journal of Rheumatology 41(2):206-15.

Rio P. Cochrane Collaboration welcomes patient participation [17]. 1999; British Medical Journal 319(7212):788-.

Santesso N, Maxwell L, Tugwell P S, Wells G A, O'Connor A M, Judd M, et al. Knowledge transfer to clinicians and consumers by the Cochrane Musculoskeletal Group. 2006; Journal of Rheumatology 33(11):2312-8.

Santesso N, Glenton C, Rosenbaum S, Pardo J, Ciapponi A, Hill S, et al. Evaluation of a Plain Language Summary template for Cochrane Reviews [abstract]. 2007;Poster at Cochrane Colloquium 68-9.

Schopler E. Collaboration between research professional and consumer. 1996; Journal of Autism and Developmental Disorders 26(2):277-80.

Tallon D, Chard J, Dieppe P. Consumer involvement in research is essential. 2000; BMJ 320(7231):380-1.

Welsh E, Jahnke N, Remmington T, Smyth A, Cates C. 20 Years of cochrane glancing backwards - moving ahead: A tale of two cochrane review groups. 2013; Paediatric Respiratory Reviews 14(3):165-7.

White P J. Evidence-based medicine for consumers: a role for the Cochrane Collaboration. 2002; Journal of the Medical Library Association 90(2):218-22.

**Unobtainable studies**

Richter, B.; Bandeira-Echtler, E. Patient participation in the Cochrane Collaboration: How can patients/consumers actively participate in a worldwide Cochrane Review Group?

Zeitschrift fur Arztliche Fortbildung und Qualitatssicherung 2005;99(6):373-375

Weingart, O.; Skoetz, N.; Lang, B.; Richter, B.; Engert, A.

Patient participation in the Cochrane Collaboration - Barriers, experience, and concepts in Germany.

Zeitschrift fur Arztliche Fortbildung und Qualitatssicherung 2005;99(6):367-371
